# Novel asymmetric representation method for solving the higher-order Ginzburg-Landau equation

**DOI:** 10.1038/srep24613

**Published:** 2016-04-18

**Authors:** Pring Wong, Lihui Pang, Ye Wu, Ming Lei, Wenjun Liu

**Affiliations:** 1 State Key Laboratory of Information Photonics and Optical Communications, Beijing University of Posts and Telecommunications, Beijing 100876, China; 2 School of Science, Beijing University of Posts and Telecommunications, P.O. Box 122, Beijing 100876, China; 3 Beijing National Laboratory for Condensed Matter Physics, Institute of Physics, Chinese Academy of Sciences, Beijing 100190, China

## Abstract

In ultrafast optics, optical pulses are generated to be of shorter pulse duration, which has enormous significance to industrial applications and scientific research. The ultrashort pulse evolution in fiber lasers can be described by the higher-order Ginzburg-Landau (GL) equation. However, analytic soliton solutions for this equation have not been obtained by use of existing methods. In this paper, a novel method is proposed to deal with this equation. The analytic soliton solution is obtained for the first time, and is proved to be stable against amplitude perturbations. Through the split-step Fourier method, the bright soliton solution is studied numerically. The analytic results here may extend the integrable methods, and could be used to study soliton dynamics for some equations in other disciplines. It may also provide the other way to obtain two-soliton solutions for higher-order GL equations.

Investigations on solitons have been made great progress since the first report on inverse scattering transformation (IST) method for soliton solutions^1^. Among them, one of active subjects is the study on optical solitons in nonlinear optics governed by nonlinear Schrödinger (NLS) equations^2^,^3^. Optical solitons can maintain their shapes and velocities during their propagation under the balance between group-velocity dispersion (GVD) and Kerr nonlinearity^4^. By virtue of the advantage of shape preserving, optical solitons have been applied in the optical switching, phase shifter, amplifier, and information storage^5^,^6^,^7^,^8^.

On the other hand, soliton solutions have been obtained in such nonlinear partial differential equations as NLS equation, Sine-Gordon equation, Gross-Pitaevskii equation, Korteweg-de Vries equation, Burgers equation, Kadomtsev-Petviashvili equation and so on^9^,^10^,^11^,^12^,^13^. Recently, the integrable nonlocal NLS equation with parity-time (PT) symmetry has been introduced and solved by the IST method^14^. In addition to the IST method, there are some other integrable methods, such as Backlünd transformations, bilinear method, separation variable method and Darboux transformation, can be used to solve those equations^15^,^16^,^17^. Among all those methods, the bilinear method may be more direct and effective to solve integrable equations.

The evolution of ultrashort pulses in fiber lasers can be described by the higher-order GL equation in the following form^18^:


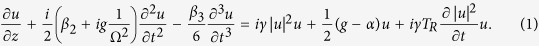


Here, *u*(*z*, *t*) is the slowly varying envelope amplitude of the pulse envelop, *z* and *t* are the propagation distance and co-moving time, respectively. The physical parameters *β*_2_, *g*, Ω, *β*_3_, *γ*, *α* and *T*_*R*_ correspond to the GVD, optical gain, gain bandwidth, third-order dispersion (TOD), Kerr nonlinearity, optical loss and intra-pulse Raman scattering, respectively. In contrast with the integrable equations mentioned above, the complex GL equation, which is non-integrable, can not be solved by the bilinear method. Owing to the modified bilinear method, one-soliton solutions for the standard form of the complex GL equation can be obtained^19^,^20^. However, for Eq. (1), the modified bilinear form of the third-order dispersion with the dependent variable transformation *u*(*z*, *t*) = *g*(*z*, *t*)/*f*(*z*, *t*)^1+*ia*^ is complicated, which has the following form:


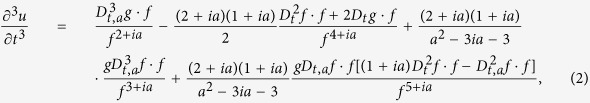


where *g*(*z*, *t*) is a complex differentiable function, *f*(*z*, *t*) is assumed to be real, and *a* is a modified parameter. The bilinear operator 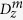 and 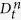 are a trivial case of modified Hirota bilinear operators, which can be defined by^19^


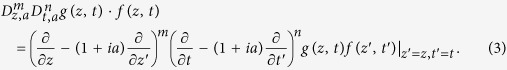


For some symbolic calculations, the term containing *f* ^5+*ia*^ in bilinear form (2) can not be merged with other items in Eq. (1). Thus, Eq. (1) is difficult to be separated into several parts by the bilinear method, and has not been solved to obtain any analytic soliton solutions with any existing methods from the known literatures.

According to the above mentioned problems, we will propose a novel method to deal with the higher-order GL equation, such as Eq. (1). This method will be built on the asymmetry of the bilinear operator directly, and will offer more freedoms and possibilities for variation than the bilinear method. A bright soliton solution for Eq. (1) will be first obtained, which is stable against amplitude perturbations. Through the split-step Fourier method, the bright soliton will be studied numerically.

## Results

### Asymmetric representation

To introduce an asymmetric function with the asymmetric parameter *a*


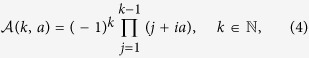


where *k* is an asymmetric degree. When the asymmetry is absent or the system is conservative, i.e. *a* = 0, the asymmetric function becomes factorial function 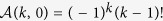. When the asymmetric degree is zero, we set 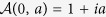 for the sake of simplicity of the asymmetric operator. When the asymmetric degree is unit, we set 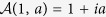 to keep the continuity of the asymmetric function. The factorial form here could assure the channel representation of the trilinear operator, which will be discussed below. Furthermore, for the asymmetric situation, we can define an asymmetric operator through the bilinear operator and the corresponding modified version


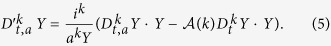


Here, *Y* is a state function of variable *t*. The asymmetric operator can be considered as an asymmetric remainder when the modified bilinear operator eliminates the regular bilinear operator.

We can deduce three linear asymmetric operators as


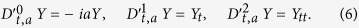


While the asymmetric degree equaling to 3, the asymmetric operator is nonlinear


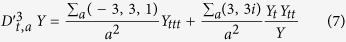


with the simplified marks


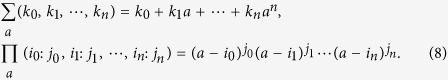


The linear asymmetric operators have a simple linear representation of differentiable functions. It indicates that the symmetry of the conventional bilinear method is not necessary for solvability, which attributes to the asymmetric operator represented by the conventional bilinear operators. The nonlinear asymmetric operator can be generalized to a bilinear form to transfer into an advanced linearity.

To generalize the nonlinear asymmetric operator, we construct a new multiplication rule


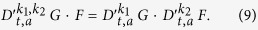


Here, 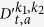 is a double-channel bilinear asymmetric (DCBA) operator. *G* and *F* are state functions. The symmetry in the bilinear method is broken. The new bilinear forms are more free and generalized, and contain the symmetric situation. The asymmetric degrees of two states can be exchanged. According to the Eq. (7), the third-order differential function can be written as


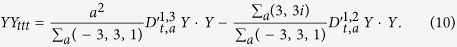


Moreover, if the state function *Y*(*t*) can be considered as the probability of two independent states, then it is equal to the product of two states’ probabilities. Let us denote as *Y* = *GF*. Then we can get a single-channel bilinear asymmetric (SCBA) operator 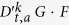 easily from Eq. (5) under linear cases. In nonlinear cases, we should define the right part of Eq. (5) without the reciprocal variable *Y*. For the simple case as the asymmetric degree equaling to 2, we can obtain the relation between SCBA and DCBA operators:


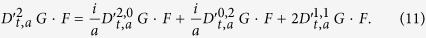


### Multilinear operators

Now we consider the asymmetric representation of the conventional bilinear operator through SCBA and DCBA operators. In general, we define a series of multilinear operators as


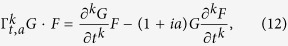


where 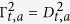. After some symbolic calculations, we obtain the bilinear asymmetric representations of the multilinear operators as


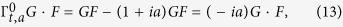



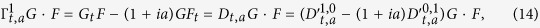



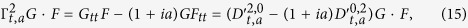



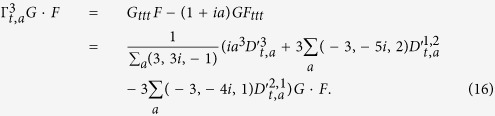


For the case of 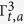, it is so complex due to the nonlinear expression of *Y*_*ttt*_. The low-order operators including 0, 1 and 2 order fit to the traditional bilinear method. However, the trilinear operator can not be written as a bilinear symmetric representation, but as the asymmetric case. The third-order dispersion term is usually presented in the dissipative situation.

So far, the bilinear asymmetric representation is more general than the symmetric representation. It can deal with the dissipative case as well as the conservative one. In the following, we will present a solvable theorem to find some interesting structures in the bilinear asymmetric equations.

### Solvable Theorem

The following low-order real coefficient equation has one-soliton solution under some appropriate conditions


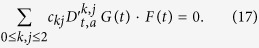


### Prove

In general, Eq. (17) is a part of the bilinear equations. With the same assumption in the conventional bilinear method, *G*(*t*) and *F*(*t*) can be written as *G*(*t*) = *εG*_1_(*t*) and *F*(*t*) = 1 + *ε*^2^*F*_2_(*t*). *ε* is a formal expansion parameter. As a bright stationary soliton solution, the form of *G*(*t*) can be set as *G*(*t*) = *e*^*wt*+*θ*^. Here, *w* and *θ* are real numbers. Substituting them into Eq. (17), we extract different power of *ε*, and get


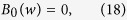



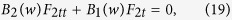



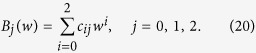


We can obtain


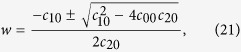



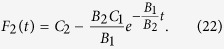


Here, *C*_1_ and *C*_2_ are integrable constants, and satisfying 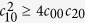. The soliton solution can be written as


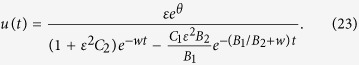


One group condition for the existence of soliton is


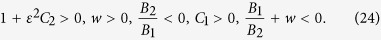


**Figure 1:**
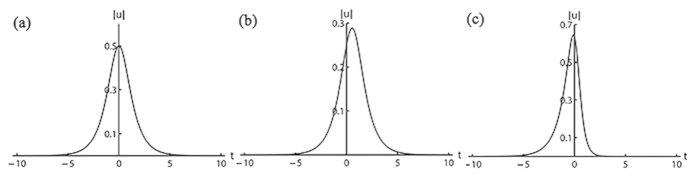
Soliton profiles of different cases. (**a**) *w* = 1, *ε* = 1, *C*1 = 2, *C*2 = 0, *B*1/*B*2 = −2; (**b**) *w* = 1, *ε* = 1, *C*1 = 2, *C*2 = 2, *B*1/*B*2 = −2; (**c**) *w* = 1, *ε* = 1, *C*1 = 2, *C*2 = 0, *B*1/*B*2 = −3.5.

If 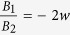, the soliton is in the sech form. Otherwise, the soliton is asymmetric. Even more, if the equation contains another variable, it will be more free to obtain one-soliton solution. The structure of the equation has soliton solutions without the bilinear symmetric representation, which extends the integrable structures. For the special values of parameters, we can show the soliton profiles in Fig. 1.

### Analytic one-soliton solution for the higher-order GL equation

With the general dependent variable transformation *u* = *G*/*F*^1+*ia*^ discussed in section 1, we substitute it into Eq. (1), and expand them directly. Based on different powers of *F*, the equation can be separated into four parts. According to the method of section 2, the asymmetric representation of Eq. (1) can be derived as


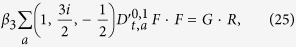



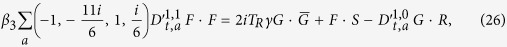



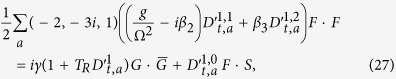



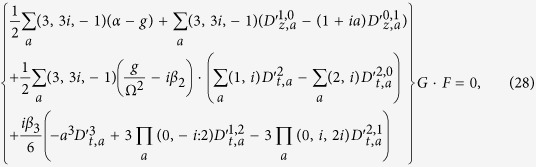


with auxiliary complex functions *R*(*z*, *t*) and *S*(*z*, *t*). The auxiliary functions here can hold part information of the equation. In the conventional bilinear method, the equation is separated into several parts irrelevantly, thus the whole information is lost without the connected auxiliary functions. In addition, the asymmetric representation can not be written as a symmetric bilinear representation.

Furthermore, a classic bright soliton solution can be assumed in the following forms,


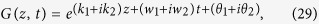



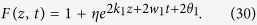


Here, *k*_1_ and *k*_2_ are parameters of the complex wave vector. *w*_1_ and *w*_2_ are complex frequencies. *θ*_1_ and *θ*_2_ are initial phases. Through substituting these assumptions into Eq. (25), we can solve the representation of function *R*(*z*, *t*) as follows,


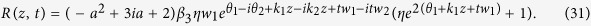


Furthermore, we can obtain the representation of function *S*(*z*, *t*) by solving Eq. (26) when *R*(*z*, *t*) has been solved. The solution of *S*(*z*, *t*) can be written as,


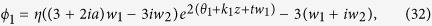



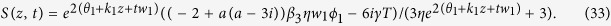


We substitute all above relations into Eq. (27), and extract the coefficients of different exponent functions. The coefficient extractions should be equal to zero to satisfy Eq. (27). At first, we extract the constant coefficients, and set it to zero. Then, we can solve the intrapulse Raman scattering coefficient,


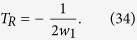


Moreover, we extract the coefficients of 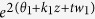, and separate it into two individual equations according to the real and imaginary parts. We can obtain the relations between the group velocity dispersion and third-order dispersion as follows,


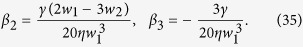


Through extracting the coefficients of 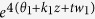, we can separate it into two equations based on real and imaginary parts. Thus, the gain width and modified parameter are solved as follows,


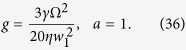


Here, the asymmetric parameter *a* is equal to 1. Finally, we can extract the coefficients of Eq. (28) to obtain the wave vector, parameter *η* and imaginary frequency. For 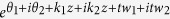, we can solve the wave vector according to real and imaginary parts,


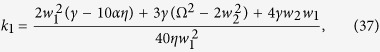



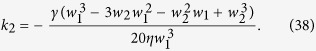


For 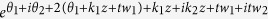, we can solve the imaginary frequency and parameter *η* as follows,


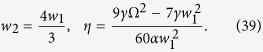


Although the form of *F* is more free to set, it is assumed to fit the bright soliton solution here. Note that the soliton solution is etric profile due to the form of *F*. The free parameters are *w*_1_, Ω, *α* and *γ*. The existence conditions for a bright soliton are *η* > 0 and *g* > 0, which require that 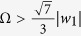. Using above constraints, the bright soliton solution can be written as


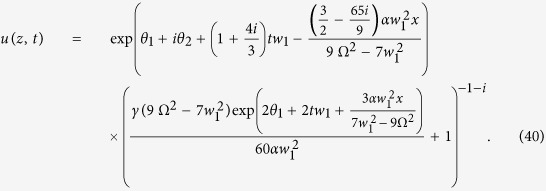


**Figure 2:**
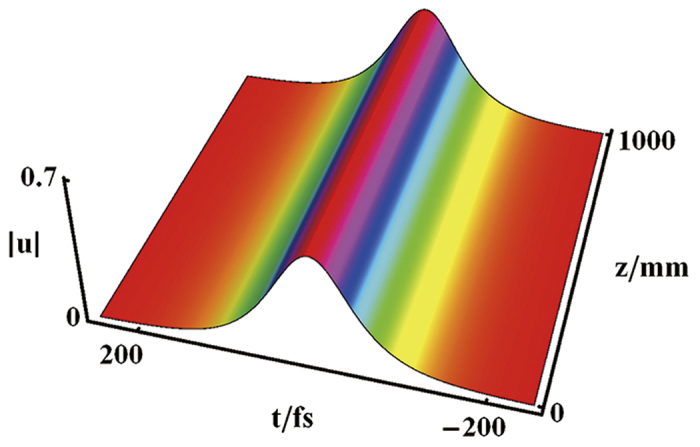
Bright soliton evolution. The appropriate parameters are chosen to be *T*_*R*_ = 20.83 *fs*, *β*_3_ = 21.15 *fs*^3^/*mm*, *β*_2_ = −0.34 *fs*^2^/*mm*, *γ* = 0.0018 *W*^−1^/*mm*, *g* = 0.0018 *dB*/*mm*, *α* = 0.0016 *dB*/*mm*, Ω = 60*μm*, *w*_1_ = −0.4  Ω, *θ*_1_ = 0, and *θ*_2_ = 0.

We select a series of physical parameters as *T*_*R*_ = 20.83 *fs*, *β*_3_ = 21.15 *fs*^3^/*mm*, *β*_2_ = −0.34 *fs*^2^/*mm*, *γ* = 0.0018 *W*^−1^/*mm*, *g* = 0.0018 *dB*/*mm*, *α* = 0.0016 *dB*/*mm*, and Ω = 60*μm*. Other parameters satisfy *w*_1_ = −0.4  Ω, *θ*_1_ = 0 and *θ*_2_ = 0. The bright soliton evolution is exhibited as shown in Fig. 2. Besides, some ordinary phenomena, e.g. phase shift, amplification and compression, can be realized by modulating the related parameters^21^.

### Numerical simulations

**Figure 3:**
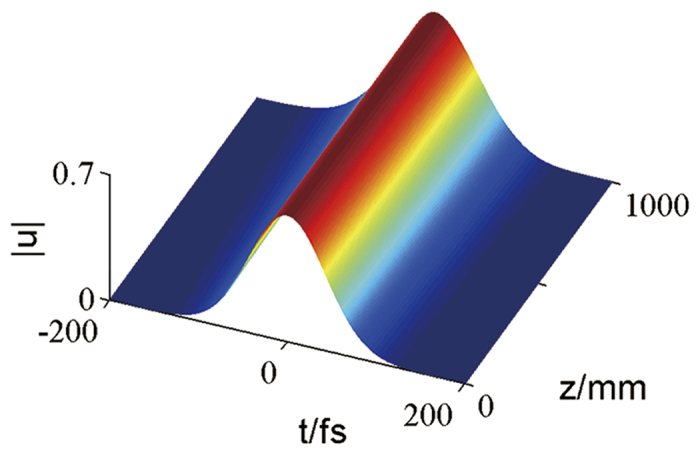
Numerical simulation of the bright soliton. The appropriate parameters are chosen to be the same as Fig. 2.

Through the split-step Fourier method^4^, we can numerically stimulate the bright soliton evolution as shown in Fig. 3. The soliton drift is due to the interaction between the third-order dispersion (TOD) and intrapulse Raman scattering. While the amplitude is perturbed by 10%, the soliton is stable still.

## Conclusion

The asymmetric representation method has been put forward to handle the analytic bright soliton solution of higher-order GL equation (1). The intrinsic structures of equations have been asymmetric, which are more general than the symmetric cases. A series concepts and methods of asymmetric representation theory have been represented. An asymmetric function has been proposed, and asymmetric operators have been constructed. Some linear operators have been presented. Furthermore, the double-channel operator has been defined, and used to make the representation of the single-channel operator. The conventional bilinear operators have been generalized to more cases, and represented by the channel operators. A solvable theorem about the structure of the asymmetric operator equation has been proved, and we have found an asymmetric structure. Through the novel asymmetric bilinear method, we have obtained a bright soliton solution for Eq. (1). Using the split-step Fourier method, the bright soliton has been numerically studied. The results in this paper extend the integrable methods, and the asymmetric representation method can be used to solve other equations in different physical systems so as to study the soliton dynamics. In addition, the method here may provide a new idea to study two-soliton solutions for the GL equation in the future research, which is still an unsolvable problem.

## Methods

### Split-step fourier transform method

In the numerical simulation of the propagation of a bright soliton, the split-step transform method is used to integrate the higher-order GL equation [Eq. (1)]. The main thought of this method is to separate the simultaneous interaction between dispersive and linear effects into series with small steps. It is useful to write Eq. (1) formally in the form


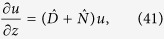


where 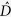 is a differential operator that accounts for dispersion and losses, and 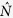 is a nonlinear operator that governs the effects of fiber nonlinearities. These operators are given by


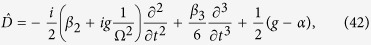



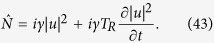


For a pulse propagates at distance z, the nonlinearity acts alone in the first half of the step *dz*/2, we only consider the effect of the linear operator


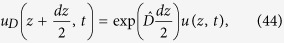


and then the nonlinear operator interacts in the whole step *dz*


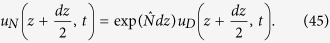


Finally, in the second half of the step *dz*/2, we consider the linear operator again, and the envelope amplitude *u*(*z* + *dz*, *t*) can be obtained as


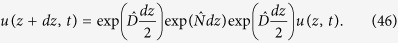


## Additional Information

**How to cite this article**: Wong, P. *et al*. Novel asymmetric representation method for solving the higher-order Ginzburg-Landau equation. *Sci. Rep*. **6**, 24613; doi: 10.1038/srep24613 (2016).
